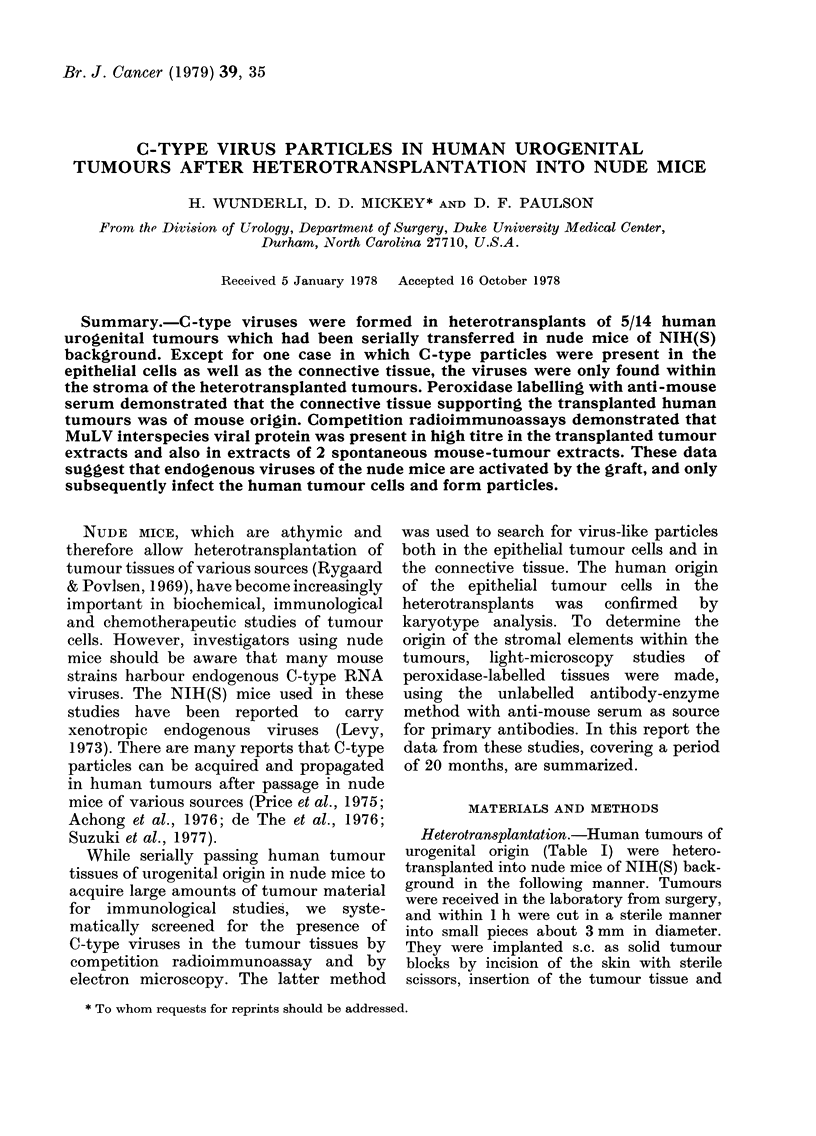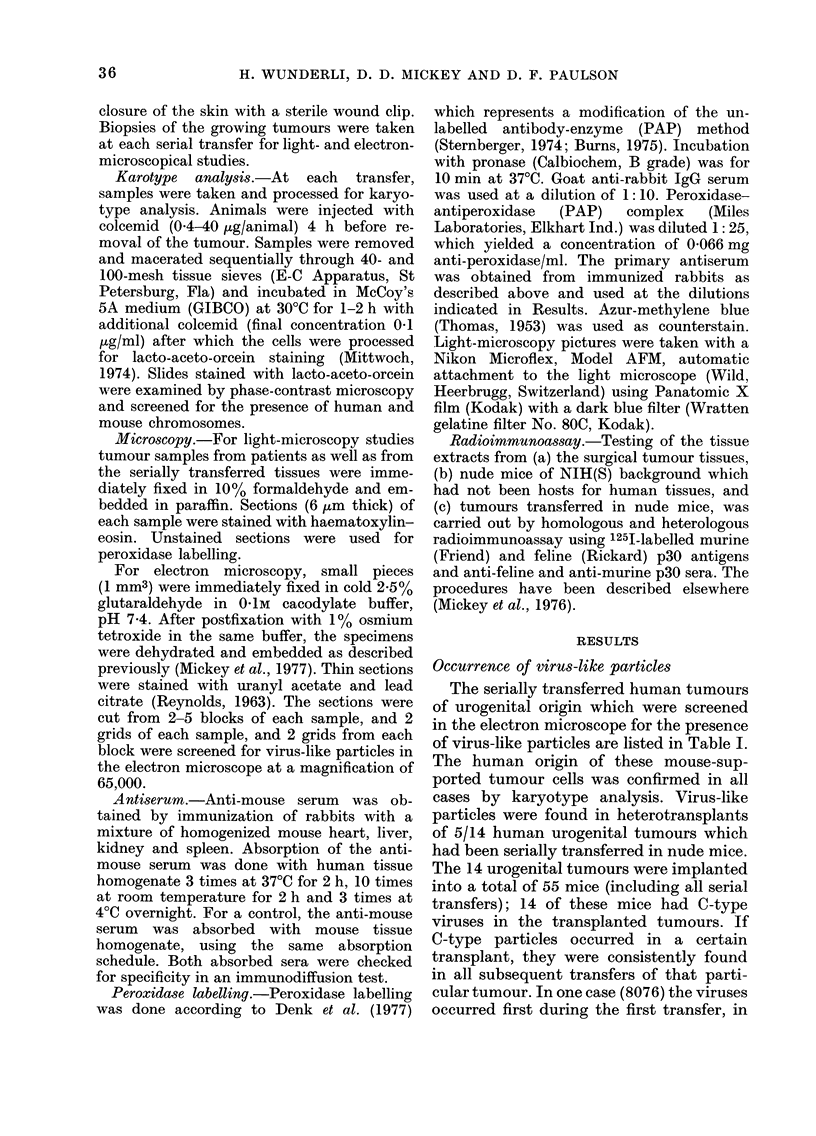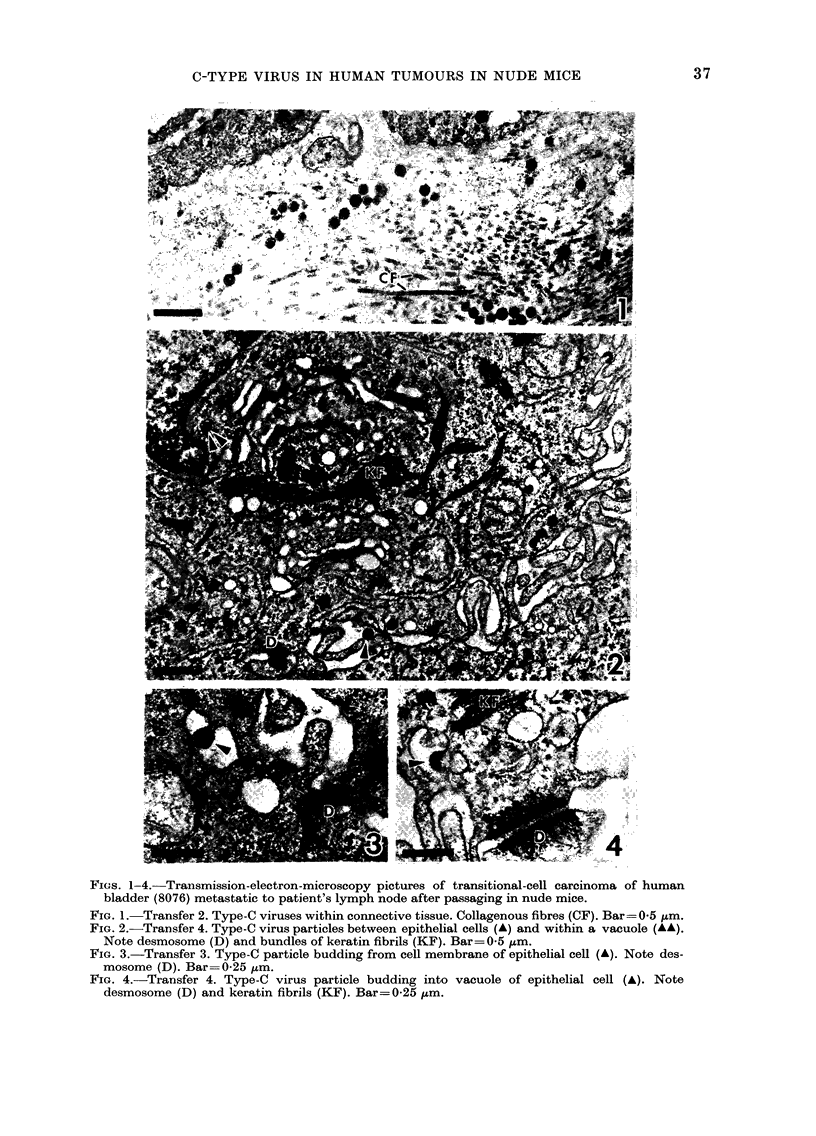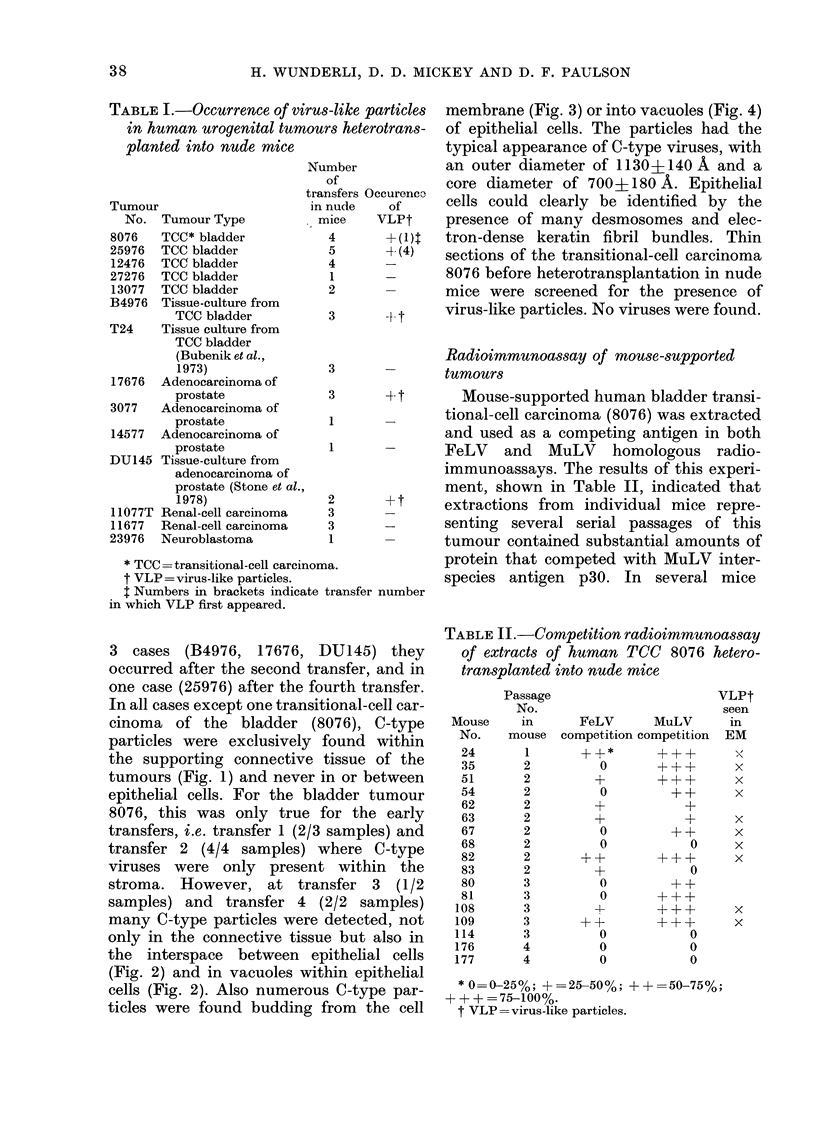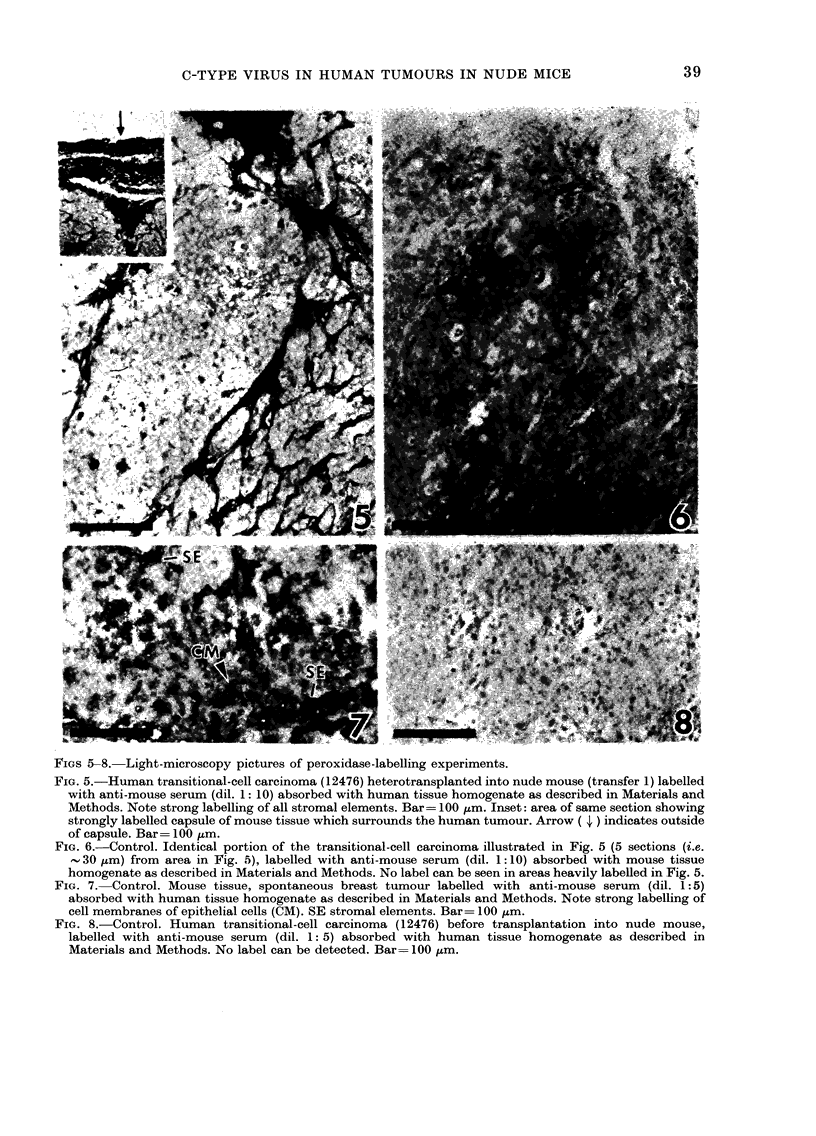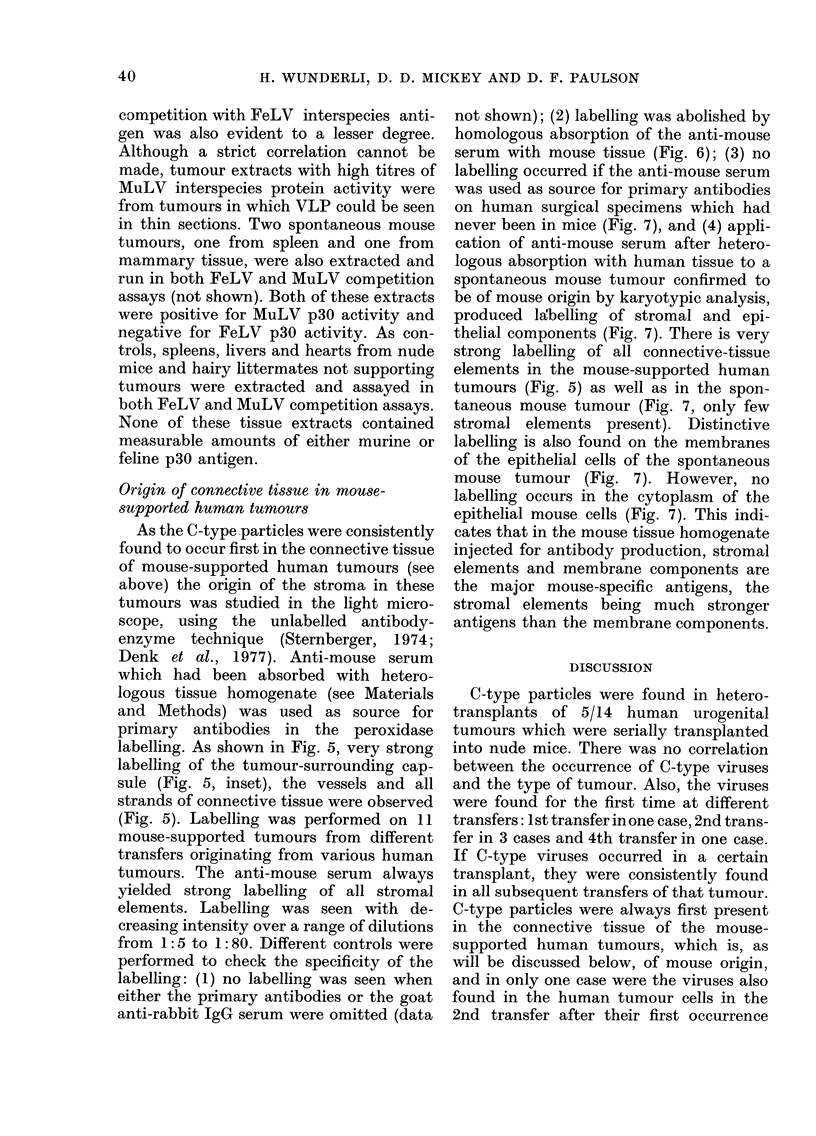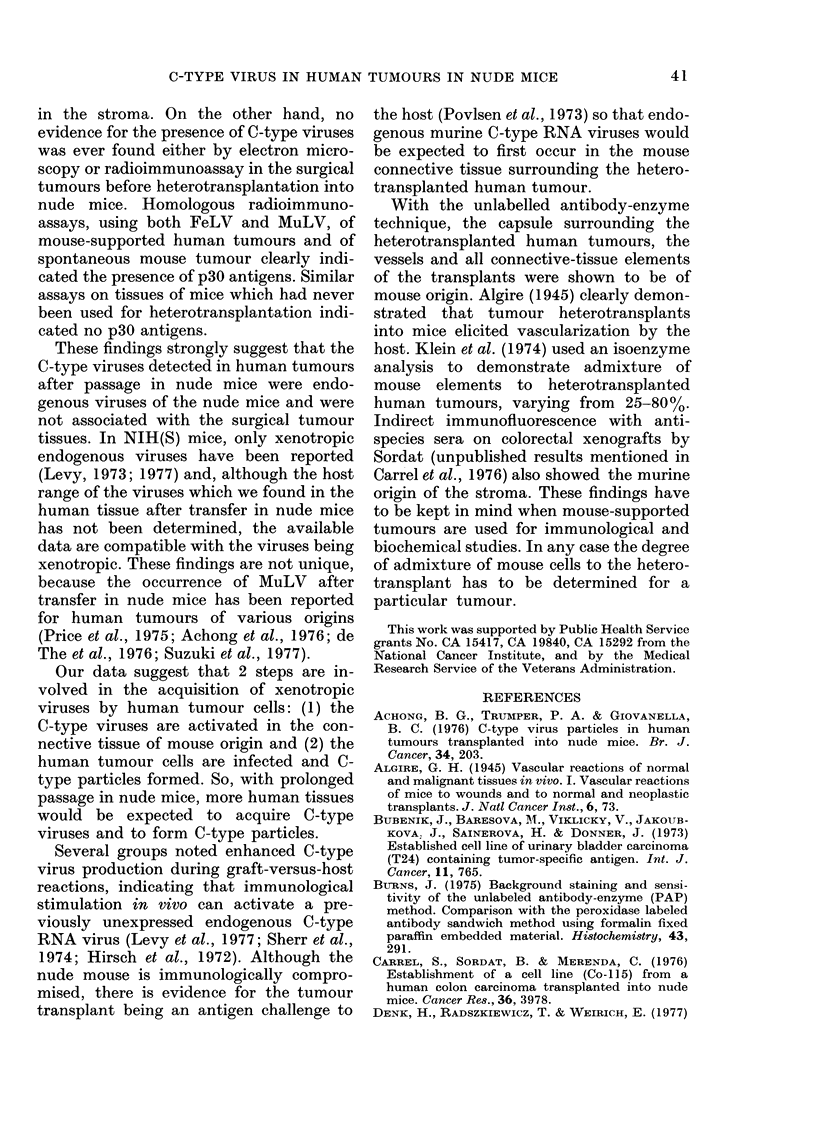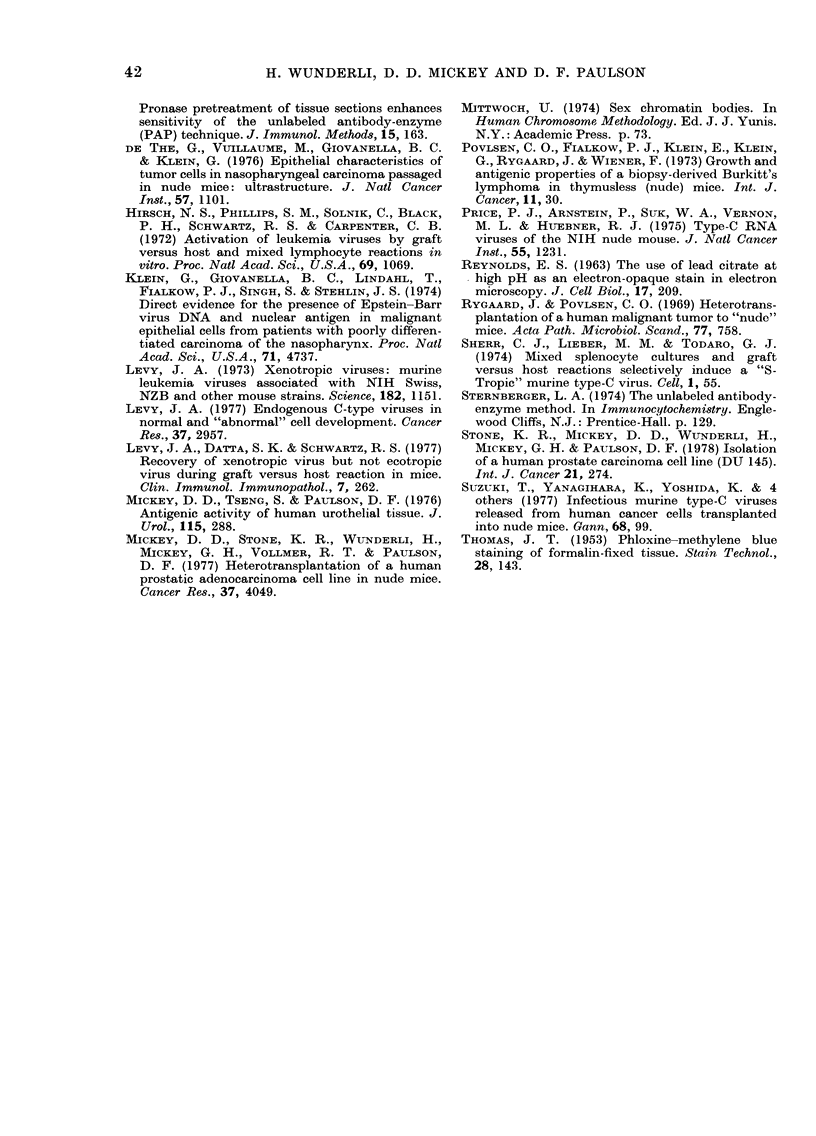# C-type virus particles in human urogenital tumours after heterotransplantation into nude mice.

**DOI:** 10.1038/bjc.1979.5

**Published:** 1979-01

**Authors:** H. Wunderli, D. D. Mickey, D. F. Paulson

## Abstract

**Images:**


Br. J. Cancer (1979) 39, 35

### C-TYPE VIRUS PARTICLES IN HUMAN UROGENITAL TUMOURS AFTER HETEROTRANSPLANTATION INTO NUDE MICE

H. WUNDERLI, D. D. MICKEY* AND D. F. PAULSON

*From the Division of Urology, Department of Surgery, Duke University Medical Center, Durham, North Carolina 27710, U.S.A.*

Received 5 January 1978 Accepted 16 October 1978

**Summary.—C-type viruses were formed in heterotransplants of 5/14 human urogenital tumours which had been serially transferred in nude mice of NIH(S) background. Except for one case in which C-type particles were present in the epithelial cells as well as the connective tissue, the viruses were only found within the stroma of the heterotransplanted tumours. Peroxidase labelling with anti-mouse serum demonstrated that the connective tissue supporting the transplanted human tumours was of mouse origin. Competition radioimmunoassays demonstrated that MuLV interspecies viral protein was present in high titre in the transplanted tumour extracts and also in extracts of 2 spontaneous mouse-tumour extracts. These data suggest that endogenous viruses of the nude mice are activated by the graft, and only subsequently infect the human tumour cells and form particles.**

NUDE MICE, which are athymic and therefore allow heterotransplantation of tumour tissues of various sources (Rygaard & Povlsen, 1969), have become increasingly important in biochemical, immunological and chemotherapeutic studies of tumour cells. However, investigators using nude mice should be aware that many mouse strains harbour endogenous C-type RNA viruses. The NIH(S) mice used in these studies have been reported to carry xenotropic endogenous viruses (Levy, 1973). There are many reports that C-type particles can be acquired and propagated in human tumours after passage in nude mice of various sources (Price *et al.*, 1975; Achong *et al.*, 1976; de The *et al.*, 1976; Suzuki *et al.*, 1977).

While serially passing human tumour tissues of urogenital origin in nude mice to acquire large amounts of tumour material for immunological studies, we systematically screened for the presence of C-type viruses in the tumour tissues by competition radioimmunoassay and by electron microscopy. The latter method was used to search for virus-like particles both in the epithelial tumour cells and in the connective tissue. The human origin of the epithelial tumour cells in the heterotransplants was confirmed by karyotype analysis. To determine the origin of the stromal elements within the tumours, light-microscopy studies of peroxidase-labelled tissues were made, using the unlabelled antibody-enzyme method with anti-mouse serum as source for primary antibodies. In this report the data from these studies, covering a period of 20 months, are summarized.

### MATERIALS AND METHODS

*Heterotransplantation.*—Human tumours of urogenital origin (Table I) were heterotransplanted into nude mice of NIH(S) background in the following manner. Tumours were received in the laboratory from surgery, and within 1 h were cut in a sterile manner into small pieces about 3 mm in diameter. They were implanted s.c. as solid tumour blocks by incision of the skin with sterile scissors, insertion of the tumour tissue and

* To whom requests for reprints should be addressed.

closure of the skin with a sterile wound clip. Biopsies of the growing tumours were taken at each serial transfer for light- and electron-microscopical studies.

*Karotype analysis.*—At each transfer, samples were taken and processed for karyotype analysis. Animals were injected with colcemid (0·4–40 μg/animal) 4 h before removal of the tumour. Samples were removed and macerated sequentially through 40- and 100-mesh tissue sieves (E-C Apparatus, St Petersburg, Fla) and incubated in McCoy's 5A medium (GIBCO) at 30°C for 1–2 h with additional colcemid (final concentration 0·1 μg/ml) after which the cells were processed for lacto-aceto-orcein staining (Mittwoch, 1974). Slides stained with lacto-aceto-orcein were examined by phase-contrast microscopy and screened for the presence of human and mouse chromosomes.

*Microscopy.*—For light-microscopy studies tumour samples from patients as well as from the serially transferred tissues were immediately fixed in 10% formaldehyde and embedded in paraffin. Sections (6 μm thick) of each sample were stained with haematoxylin–eosin. Unstained sections were used for peroxidase labelling.

For electron microscopy, small pieces (1 mm$^3$) were immediately fixed in cold 2·5% glutaraldehyde in 0·1M cacodylate buffer, pH 7·4. After postfixation with 1% osmium tetroxide in the same buffer, the specimens were dehydrated and embedded as described previously (Mickey *et al.*, 1977). Thin sections were stained with uranyl acetate and lead citrate (Reynolds, 1963). The sections were cut from 2–5 blocks of each sample, and 2 grids of each sample, and 2 grids from each block were screened for virus-like particles in the electron microscope at a magnification of 65,000.

*Antiserum.*—Anti-mouse serum was obtained by immunization of rabbits with a mixture of homogenized mouse heart, liver, kidney and spleen. Absorption of the anti-mouse serum was done with human tissue homogenate 3 times at 37°C for 2 h, 10 times at room temperature for 2 h and 3 times at 4°C overnight. For a control, the anti-mouse serum was absorbed with mouse tissue homogenate, using the same absorption schedule. Both absorbed sera were checked for specificity in an immunodiffusion test.

*Peroxidase labelling.*—Peroxidase labelling was done according to Denk *et al.* (1977) which represents a modification of the unlabelled antibody-enzyme (PAP) method (Sternberger, 1974; Burns, 1975). Incubation with pronase (Calbiochem, B grade) was for 10 min at 37°C. Goat anti-rabbit IgG serum was used at a dilution of 1:10. Peroxidase–antiperoxidase (PAP) complex (Miles Laboratories, Elkhart Ind.) was diluted 1:25, which yielded a concentration of 0·066 mg anti-peroxidase/ml. The primary antiserum was obtained from immunized rabbits as described above and used at the dilutions indicated in Results. Azur-methylene blue (Thomas, 1953) was used as counterstain. Light-microscopy pictures were taken with a Nikon Microflex, Model AFM, automatic attachment to the light microscope (Wild, Heerbrugg, Switzerland) using Panatomic X film (Kodak) with a dark blue filter (Wratten gelatine filter No. 80C, Kodak).

*Radioimmunoassay.*—Testing of the tissue extracts from (a) the surgical tumour tissues, (b) nude mice of NIH(S) background which had not been hosts for human tissues, and (c) tumours transferred in nude mice, was carried out by homologous and heterologous radioimmunoassay using $^{125}$I-labelled murine (Friend) and feline (Rickard) p30 antigens and anti-feline and anti-murine p30 sera. The procedures have been described elsewhere (Mickey *et al.*, 1976).

### RESULTS

### *Occurrence of virus-like particles*

The serially transferred human tumours of urogenital origin which were screened in the electron microscope for the presence of virus-like particles are listed in Table I. The human origin of these mouse-supported tumour cells was confirmed in all cases by karyotype analysis. Virus-like particles were found in heterotransplants of 5/14 human urogenital tumours which had been serially transferred in nude mice. The 14 urogenital tumours were implanted into a total of 55 mice (including all serial transfers); 14 of these mice had C-type viruses in the transplanted tumours. If C-type particles occurred in a certain transplant, they were consistently found in all subsequent transfers of that particular tumour. In one case (8076) the viruses occurred first during the first transfer, in

FIGS. 1–4.—Transmission-electron-microscopy pictures of transitional-cell carcinoma of human bladder (8076) metastatic to patient's lymph node after passaging in nude mice.

FIG. 1.—Transfer 2. Type-C viruses within connective tissue. Collagenous fibres (CF). Bar = 0·5 μm.

FIG. 2.—Transfer 4. Type-C virus particles between epithelial cells (▲) and within a vacuole (▲▲). Note desmosome (D) and bundles of keratin fibrils (KF). Bar = 0·5 μm.

FIG. 3.—Transfer 3. Type-C particle budding from cell membrane of epithelial cell (▲). Note desmosome (D). Bar = 0·25 μm.

FIG. 4.—Transfer 4. Type-C virus particle budding into vacuole of epithelial cell (▲). Note desmosome (D) and keratin fibrils (KF). Bar = 0·25 μm.

TABLE I.—*Occurrence of virus-like particles in human urogenital tumours heterotransplanted into nude mice*

| Tumour No. | Tumour Type | Number of transfers in nude mice | Occurence of VLP† |
|---|---|---|---|
| 8076 | TCC* bladder | 4 | +(1)‡ |
| 25976 | TCC bladder | 5 | +(4) |
| 12476 | TCC bladder | 4 | — |
| 27276 | TCC bladder | 1 | — |
| 13077 | TCC bladder | 2 | — |
| B4976 | Tissue-culture from TCC bladder | 3 | +† |
| T24 | Tissue culture from TCC bladder (Bubenik *et al.*, 1973) | 3 | — |
| 17676 | Adenocarcinoma of prostate | 3 | +† |
| 3077 | Adenocarcinoma of prostate | 1 | — |
| 14577 | Adenocarcinoma of prostate | 1 | — |
| DU145 | Tissue-culture from adenocarcinoma of prostate (Stone *et al.*, 1978) | 2 | +† |
| 11077T | Renal-cell carcinoma | 3 | — |
| 11677 | Renal-cell carcinoma | 3 | — |
| 23976 | Neuroblastoma | 1 | — |

* TCC = transitional-cell carcinoma.
† VLP = virus-like particles.
‡ Numbers in brackets indicate transfer number in which VLP first appeared.

3 cases (B4976, 17676, DU145) they occurred after the second transfer, and in one case (25976) after the fourth transfer. In all cases except one transitional-cell carcinoma of the bladder (8076), C-type particles were exclusively found within the supporting connective tissue of the tumours (Fig. 1) and never in or between epithelial cells. For the bladder tumour 8076, this was only true for the early transfers, *i.e.* transfer 1 (2/3 samples) and transfer 2 (4/4 samples) where C-type viruses were only present within the stroma. However, at transfer 3 (1/2 samples) and transfer 4 (2/2 samples) many C-type particles were detected, not only in the connective tissue but also in the interspace between epithelial cells (Fig. 2) and in vacuoles within epithelial cells (Fig. 2). Also numerous C-type particles were found budding from the cell membrane (Fig. 3) or into vacuoles (Fig. 4) of epithelial cells. The particles had the typical appearance of C-type viruses, with an outer diameter of 1130±140 Å and a core diameter of 700±180 Å. Epithelial cells could clearly be identified by the presence of many desmosomes and electron-dense keratin fibril bundles. Thin sections of the transitional-cell carcinoma 8076 before heterotransplantation in nude mice were screened for the presence of virus-like particles. No viruses were found.

*Radioimmunoassay of mouse-supported tumours*

Mouse-supported human bladder transitional-cell carcinoma (8076) was extracted and used as a competing antigen in both FeLV and MuLV homologous radioimmunoassays. The results of this experiment, shown in Table II, indicated that extractions from individual mice representing several serial passages of this tumour contained substantial amounts of protein that competed with MuLV interspecies antigen p30. In several mice

TABLE II.—*Competition radioimmunoassay of extracts of human TCC 8076 heterotransplanted into nude mice*

| Mouse No. | Passage No. in mouse | FeLV competition | MuLV competition | VLP† seen in EM |
|---|---|---|---|---|
| 24 | 1 | ++* | +++ | × |
| 35 | 2 | 0 | +++ | × |
| 51 | 2 | + | +++ | × |
| 54 | 2 | 0 | ++ | × |
| 62 | 2 | + | + | |
| 63 | 2 | + | + | × |
| 67 | 2 | 0 | ++ | × |
| 68 | 2 | 0 | 0 | × |
| 82 | 2 | ++ | +++ | × |
| 83 | 2 | + | 0 | |
| 80 | 3 | 0 | ++ | |
| 81 | 3 | 0 | +++ | |
| 108 | 3 | + | +++ | × |
| 109 | 3 | ++ | +++ | × |
| 114 | 3 | 0 | 0 | |
| 176 | 4 | 0 | 0 | |
| 177 | 4 | 0 | 0 | |

* 0 = 0–25%; + = 25–50%; ++ = 50–75%; +++ = 75–100%.

† VLP = virus-like particles.

FIGS 5–8.—Light-microscopy pictures of peroxidase-labelling experiments.

FIG. 5.—Human transitional-cell carcinoma (12476) heterotransplanted into nude mouse (transfer 1) labelled with anti-mouse serum (dil. 1: 10) absorbed with human tissue homogenate as described in Materials and Methods. Note strong labelling of all stromal elements. Bar = 100 μm. Inset: area of same section showing strongly labelled capsule of mouse tissue which surrounds the human tumour. Arrow ( ↓ ) indicates outside of capsule. Bar = 100 μm.

FIG. 6.—Control. Identical portion of the transitional-cell carcinoma illustrated in Fig. 5 (5 sections (*i.e.* ~30 μm) from area in Fig. 5), labelled with anti-mouse serum (dil. 1:10) absorbed with mouse tissue homogenate as described in Materials and Methods. No label can be seen in areas heavily labelled in Fig. 5.

FIG. 7.—Control. Mouse tissue, spontaneous breast tumour labelled with anti-mouse serum (dil. 1:5) absorbed with human tissue homogenate as described in Materials and Methods. Note strong labelling of cell membranes of epithelial cells (CM). SE stromal elements. Bar = 100 μm.

FIG. 8.—Control. Human transitional-cell carcinoma (12476) before transplantation into nude mouse, labelled with anti-mouse serum (dil. 1: 5) absorbed with human tissue homogenate as described in Materials and Methods. No label can be detected. Bar = 100 μm.

competition with FeLV interspecies antigen was also evident to a lesser degree. Although a strict correlation cannot be made, tumour extracts with high titres of MuLV interspecies protein activity were from tumours in which VLP could be seen in thin sections. Two spontaneous mouse tumours, one from spleen and one from mammary tissue, were also extracted and run in both FeLV and MuLV competition assays (not shown). Both of these extracts were positive for MuLV p30 activity and negative for FeLV p30 activity. As controls, spleens, livers and hearts from nude mice and hairy littermates not supporting tumours were extracted and assayed in both FeLV and MuLV competition assays. None of these tissue extracts contained measurable amounts of either murine or feline p30 antigen.

*Origin of connective tissue in mouse-supported human tumours*

As the C-type particles were consistently found to occur first in the connective tissue of mouse-supported human tumours (see above) the origin of the stroma in these tumours was studied in the light microscope, using the unlabelled antibody-enzyme technique (Sternberger, 1974; Denk *et al.*, 1977). Anti-mouse serum which had been absorbed with heterologous tissue homogenate (see Materials and Methods) was used as source for primary antibodies in the peroxidase labelling. As shown in Fig. 5, very strong labelling of the tumour-surrounding capsule (Fig. 5, inset), the vessels and all strands of connective tissue were observed (Fig. 5). Labelling was performed on 11 mouse-supported tumours from different transfers originating from various human tumours. The anti-mouse serum always yielded strong labelling of all stromal elements. Labelling was seen with decreasing intensity over a range of dilutions from 1:5 to 1:80. Different controls were performed to check the specificity of the labelling: (1) no labelling was seen when either the primary antibodies or the goat anti-rabbit IgG serum were omitted (data not shown); (2) labelling was abolished by homologous absorption of the anti-mouse serum with mouse tissue (Fig. 6); (3) no labelling occurred if the anti-mouse serum was used as source for primary antibodies on human surgical specimens which had never been in mice (Fig. 7), and (4) application of anti-mouse serum after heterologous absorption with human tissue to a spontaneous mouse tumour confirmed to be of mouse origin by karyotypic analysis, produced labelling of stromal and epithelial components (Fig. 7). There is very strong labelling of all connective-tissue elements in the mouse-supported human tumours (Fig. 5) as well as in the spontaneous mouse tumour (Fig. 7, only few stromal elements present). Distinctive labelling is also found on the membranes of the epithelial cells of the spontaneous mouse tumour (Fig. 7). However, no labelling occurs in the cytoplasm of the epithelial mouse cells (Fig. 7). This indicates that in the mouse tissue homogenate injected for antibody production, stromal elements and membrane components are the major mouse-specific antigens, the stromal elements being much stronger antigens than the membrane components.

### DISCUSSION

C-type particles were found in heterotransplants of 5/14 human urogenital tumours which were serially transplanted into nude mice. There was no correlation between the occurrence of C-type viruses and the type of tumour. Also, the viruses were found for the first time at different transfers: 1st transfer in one case, 2nd transfer in 3 cases and 4th transfer in one case. If C-type viruses occurred in a certain transplant, they were consistently found in all subsequent transfers of that tumour. C-type particles were always first present in the connective tissue of the mouse-supported human tumours, which is, as will be discussed below, of mouse origin, and in only one case were the viruses also found in the human tumour cells in the 2nd transfer after their first occurrence in the stroma. On the other hand, no evidence for the presence of C-type viruses was ever found either by electron microscopy or radioimmunoassay in the surgical tumours before heterotransplantation into nude mice. Homologous radioimmunoassays, using both FeLV and MuLV, of mouse-supported human tumours and of spontaneous mouse tumour clearly indicated the presence of p30 antigens. Similar assays on tissues of mice which had never been used for heterotransplantation indicated no p30 antigens.

These findings strongly suggest that the C-type viruses detected in human tumours after passage in nude mice were endogenous viruses of the nude mice and were not associated with the surgical tumour tissues. In NIH(S) mice, only xenotropic endogenous viruses have been reported (Levy, 1973; 1977) and, although the host range of the viruses which we found in the human tissue after transfer in nude mice has not been determined, the available data are compatible with the viruses being xenotropic. These findings are not unique, because the occurrence of MuLV after transfer in nude mice has been reported for human tumours of various origins (Price *et al.*, 1975; Achong *et al.*, 1976; de The *et al.*, 1976; Suzuki *et al.*, 1977).

Our data suggest that 2 steps are involved in the acquisition of xenotropic viruses by human tumour cells: (1) the C-type viruses are activated in the connective tissue of mouse origin and (2) the human tumour cells are infected and C-type particles formed. So, with prolonged passage in nude mice, more human tissues would be expected to acquire C-type viruses and to form C-type particles.

Several groups noted enhanced C-type virus production during graft-versus-host reactions, indicating that immunological stimulation *in vivo* can activate a previously unexpressed endogenous C-type RNA virus (Levy *et al.*, 1977; Sherr *et al.*, 1974; Hirsch *et al.*, 1972). Although the nude mouse is immunologically compromised, there is evidence for the tumour transplant being an antigen challenge to the host (Povlsen *et al.*, 1973) so that endogenous murine C-type RNA viruses would be expected to first occur in the mouse connective tissue surrounding the heterotransplanted human tumour.

With the unlabelled antibody-enzyme technique, the capsule surrounding the heterotransplanted human tumours, the vessels and all connective-tissue elements of the transplants were shown to be of mouse origin. Algire (1945) clearly demonstrated that tumour heterotransplants into mice elicited vascularization by the host. Klein *et al.* (1974) used an isoenzyme analysis to demonstrate admixture of mouse elements to heterotransplanted human tumours, varying from 25–80%. Indirect immunofluorescence with anti-species sera on colorectal xenografts by Sordat (unpublished results mentioned in Carrel *et al.*, 1976) also showed the murine origin of the stroma. These findings have to be kept in mind when mouse-supported tumours are used for immunological and biochemical studies. In any case the degree of admixture of mouse cells to the heterotransplant has to be determined for a particular tumour.

This work was supported by Public Health Service grants No. CA 15417, CA 19840, CA 15292 from the National Cancer Institute, and by the Medical Research Service of the Veterans Administration.

### REFERENCES

ACHONG, B. G., TRUMPER, P. A. & GIOVANELLA, B. C. (1976) C-type virus particles in human tumours transplanted into nude mice. *Br. J. Cancer*, **34**, 203.

ALGIRE, G. H. (1945) Vascular reactions of normal and malignant tissues *in vivo*. I. Vascular reactions of mice to wounds and to normal and neoplastic transplants. *J. Natl Cancer Inst.*, **6**, 73.

BUBENIK, J., BARESOVA, M., VIKLICKY, V., JAKOUBKOVA, J., SAINEROVA, H. & DONNER, J. (1973) Established cell line of urinary bladder carcinoma (T24) containing tumor-specific antigen. *Int. J. Cancer*, **11**, 765.

BURNS, J. (1975) Background staining and sensitivity of the unlabeled antibody-enzyme (PAP) method. Comparison with the peroxidase labeled antibody sandwich method using formalin fixed paraffin embedded material. *Histochemistry*, **43**, 291.

CARREL, S., SORDAT, B. & MERENDA, C. (1976) Establishment of a cell line (Co-115) from a human colon carcinoma transplanted into nude mice. *Cancer Res.*, **36**, 3978.

DENK, H., RADSZKIEWICZ, T. & WEIRICH, E. (1977)

Pronase pretreatment of tissue sections enhances sensitivity of the unlabeled antibody-enzyme (PAP) technique. *J. Immunol. Methods*, **15**, 163.

DE THE, G., VUILLAUME, M., GIOVANELLA, B. C. & KLEIN, G. (1976) Epithelial characteristics of tumor cells in nasopharyngeal carcinoma passaged in nude mice: ultrastructure. *J. Natl Cancer Inst.*, **57**, 1101.

HIRSCH, N. S., PHILLIPS, S. M., SOLNIK, C., BLACK, P. H., SCHWARTZ, R. S. & CARPENTER, C. B. (1972) Activation of leukemia viruses by graft versus host and mixed lymphocyte reactions *in vitro*. *Proc. Natl Acad. Sci., U.S.A.*, **69**, 1069.

KLEIN, G., GIOVANELLA, B. C., LINDAHL, T., FIALKOW, P. J., SINGH, S. & STEHLIN, J. S. (1974) Direct evidence for the presence of Epstein–Barr virus DNA and nuclear antigen in malignant epithelial cells from patients with poorly differentiated carcinoma of the nasopharynx. *Proc. Natl Acad. Sci., U.S.A.*, **71**, 4737.

LEVY, J. A. (1973) Xenotropic viruses: murine leukemia viruses associated with NIH Swiss, NZB and other mouse strains. *Science*, **182**, 1151.

LEVY, J. A. (1977) Endogenous C-type viruses in normal and "abnormal" cell development. *Cancer Res.*, **37**, 2957.

LEVY, J. A., DATTA, S. K. & SCHWARTZ, R. S. (1977) Recovery of xenotropic virus but not ecotropic virus during graft versus host reaction in mice. *Clin. Immunol. Immunopathol.*, **7**, 262.

MICKEY, D. D., TSENG, S. & PAULSON, D. F. (1976) Antigenic activity of human urothelial tissue. *J. Urol.*, **115**, 288.

MICKEY, D. D., STONE, K. R., WUNDERLI, H., MICKEY, G. H., VOLLMER, R. T. & PAULSON, D. F. (1977) Heterotransplantation of a human prostatic adenocarcinoma cell line in nude mice. *Cancer Res.*, **37**, 4049.

MITTWOCH, U. (1974) Sex chromatin bodies. In *Human Chromosome Methodology*. Ed. J. J. Yunis. N.Y.: Academic Press. p. 73.

POVLSEN, C. O., FIALKOW, P. J., KLEIN, E., KLEIN, G., RYGAARD, J. & WIENER, F. (1973) Growth and antigenic properties of a biopsy-derived Burkitt's lymphoma in thymusless (nude) mice. *Int. J. Cancer*, **11**, 30.

PRICE, P. J., ARNSTEIN, P., SUK, W. A., VERNON, M. L. & HUEBNER, R. J. (1975) Type-C RNA viruses of the NIH nude mouse. *J. Natl Cancer Inst.*, **55**, 1231.

REYNOLDS, E. S. (1963) The use of lead citrate at high pH as an electron-opaque stain in electron microscopy. *J. Cell Biol.*, **17**, 209.

RYGAARD, J. & POVLSEN, C. O. (1969) Heterotransplantation of a human malignant tumor to "nude" mice. *Acta Path. Microbiol. Scand.*, **77**, 758.

SHERR, C. J., LIEBER, M. M. & TODARO, G. J. (1974) Mixed splenocyte cultures and graft versus host reactions selectively induce a "S-Tropic" murine type-C virus. *Cell*, **1**, 55.

STERNBERGER, L. A. (1974) The unlabeled antibody-enzyme method. In *Immunocytochemistry*. Englewood Cliffs, N.J.: Prentice-Hall. p. 129.

STONE, K. R., MICKEY, D. D., WUNDERLI, H., MICKEY, G. H. & PAULSON, D. F. (1978) Isolation of a human prostate carcinoma cell line (DU 145). *Int. J. Cancer* **21**, 274.

SUZUKI, T., YANAGIHARA, K., YOSHIDA, K. & 4 others (1977) Infectious murine type-C viruses released from human cancer cells transplanted into nude mice. *Gann*, **68**, 99.

THOMAS, J. T. (1953) Phloxine–methylene blue staining of formalin-fixed tissue. *Stain Technol.*, **28**, 143.